# Skeletal Anomaly Monitoring in Rainbow Trout (*Oncorhynchus mykiss*, Walbaum 1792) Reared under Different Conditions

**DOI:** 10.1371/journal.pone.0096983

**Published:** 2014-05-08

**Authors:** Clara Boglione, Domitilla Pulcini, Michele Scardi, Elisa Palamara, Tommaso Russo, Stefano Cataudella

**Affiliations:** 1 Laboratory of Experimental Ecology and Aquaculture, Department of Biology, “Tor Vergata” University of Rome, Rome, Italy; 2 Council for Research in Agriculture – Animal Production Centre, Rome, Italy; Aristotle University of Thessaloniki, Greece

## Abstract

The incidence of skeletal anomalies could be used as an indicator of the “quality” of rearing conditions as these anomalies are thought to result from the inability of homeostatic mechanisms to compensate for environmentally-induced stress and/or altered genetic factors. Identification of rearing conditions that lower the rate of anomalies can be an important step toward profitable aquaculture as malformed market-size fish have to be discarded, thus reducing fish farmers’ profits. In this study, the occurrence of skeletal anomalies in adult rainbow trout grown under intensive and organic conditions was monitored. As organic aquaculture animal production is in its early stages, organic broodstock is not available in sufficient quantities. Non-organic juveniles could, therefore, be used for on-growing purposes in organic aquaculture production cycle. Thus, the adult fish analysed in this study experienced intensive conditions during juvenile rearing. Significant differences in the pattern of anomalies were detected between organically and intensively-ongrown specimens, although the occurrence of severe, commercially important anomalies, affecting 2–12.5% of individuals, was comparable in the two systems. Thus, organic aquaculture needs to be improved in order to significantly reduce the incidence of severe anomalies in rainbow trout.

## Introduction

Aquaculture of fish and other aquatic animals has grown rapidly in the last thirty years [1]. Most fish aquaculture production comes from freshwater, with salmonid farming making a significant contribution to global aquaculture production volumes [1], [2], [3]. Rainbow trout *Oncorhynchus mykiss* (Walbaum 1792) is a dominant farmed salmonids in Europe and North America [1]. Naturally distributed along the Pacific coast of North America and on the Kamchatka Peninsula [4], [5], rainbow trout has been extensively introduced for aquaculture practically all over the world since the mid-1800s.

Farmed fish are often affected by skeletal anomalies, with the incidence depending on the species, developmental stage, and rearing methodology. Skeletal anomalies may arise in captivity due to both genetic (such as inbreeding depression due to artificial selection [6]– and triploidy [9], [10]) and environmental causes [11], [12]. Rearing conditions different from the species- or developmental stage-specific ones often cause the onset of skeletal anomalies[12]–[22]. In farmed salmonids, some studies found no relationship between incidence of anomalies and captive conditions [23], [24], while others ascribed displacement of vertebral centra, fused and compressed vertebral axis,and decreased bone quality to fast-growing rearing conditions [9], [10], [25]–[28]. Among environmental causes, inappropriate rearing densities were reported in previous studies as causative factors of bone malformations [12] in Atlantic salmon (*Salmo salar*, L. 1758) fry and parr [29]. In several species of commercial interest (i.e. *Dicentrarchus labrax*, *Sparus aurata*, *Epinephelus marginatus*, *Dentex dentex*, *Pagrus pagrus*), a reduction in skeletal anomalies (especially commercially relevant ones) has been detected in semi-intensive rearing conditions, characterized by lower densities and larger volumes [11], [30]–[36]. Commercially significant anomalies affect the head and the vertebral axis, thus altering external shape and swimming/feeding performance, with consequent lower growth rate, economic value and welfare status, and higher susceptibility to stress, pathogens and bacteria [13]–[15], [37]–[41]. Seriously malformed market-size fish have to be discarded or sold at lower than market price due to the consumer’s reluctance to buy ‘bad-looking’ products.

This study tested whether any difference exists in the number (meristic counts) and shape (occurrence of anomalies) of skeletal elements in adult rainbow trout grown under intensive *vs* organic aquaculture.

## Materials and Methods

A total of 533 adult rainbow trout (which is not a protected or endangered species) were collected from five European fish farms: (1) two intensive (“Az. Agricola Troticoltura Rossi”, Abruzzo, Italy; “Az. Agricola Rio Fontane”, Veneto, Italy), denoted, respectively, as INT1 and INT2; (2) three organic, (“Az. Agricola Troticoltura Rossi”, Lazio, Italy; “Az. Agricola Rio Fontane”, Veneto, Italy; “Azienda Agricola Pura” – Switzerland) denoted, respectively, as ORG1, ORG2 and ORG3. The latter followed the standards for organic productions developed by Naturland, ECOCERT and Biosuisse certification bodies, respectively. No specific permissions were required for the activities carried out in the above-mentioned locations as they were not protected areas. The owners of the farms gave permission to collect the samples for this study. The main features (material, shape and size of the rearing ponds, temperature, water flow, density) of the farms are reported in Table 1.

**Table 1:** Features of the farms where fish were collected (organic ones in grey).

Farm	Pond	Surface	Volume	Water flow	Temperature	Density
**INT1** (Italy, Abruzzo)	Rectangular concreteraceways	800×0.7	560	50–100	10–10.5	55
**INT2** (Italy, Piedmont)	Rectangular concreteraceways	1000/1300×0.5	500–650	100	12.5	40
**ORG1** (Italy, Lazio)	Squared earth ponds	800/1300×0.8	650–1150	50–100	10.5–11	15–30*
**ORG2** (Italy, Piedmont)	Rectangularearth ponds	600/1000×0.6	300	300	10–10.5	12
**ORG3** (Switzerland)	Rectangular, vegetatedearth ponds	720×0.6	430	100	8	10–12

Surface = m^2^ ⋅ m; Volume = m^3^; Water flow = l⋅s^−1^; Temperature = °C; Density = kg⋅m^−3^. * Individuals were temporarily stocked at high densities (30 kg⋅m^−3^).

Different strains were collected in the farms: Italian, French, Spanish, Swiss and American. Italian fish were collected from all the farms except ORG3; French fish were collected from INT1, Spanish fish from INT2, American fish from ORG2 and Swiss fish from ORG3.

The number of observed specimens, the total length (TL) range, and the genetic origin of the lots are reported in Table 2.

**Table 2:** Genetic origin (*Origin*), geographic origin of the source population (*source*), number (*n*) and total length (TL mean ± standard deviation) of observed specimens.

Farm	Origin	Source	Lot	n	TL±S.D. (cm)
**INT1**	Italy	USA	1	46	28.7±3.2
	France	USA	2	193	30.1±4.4
**INT2**	Italy	USA	3	16	33.5±4.7
	Spain	France	4	32	12.6±1.1
**ORG1**	Italy	USA	5	108	31.7±3.4
**ORG2**	Italy	USA	6	29	24.7±4.4
	USA	USA	7	60	26.8±3.1
**ORG3**	Switzerland	Germany	8	49	20.2±2.7

Data referring to source populations are from [4] and [42].

A detailed description of the historical background of these strains is available in [43].

Commission Regulation (EC) No 710/2009 of 5 August 2009 states that: “Given the early stage of organic aquaculture animal production, organic broodstock is not available in sufficient quantities. Provision should be made for the introduction of non-organic broodstock and juveniles under certain conditions.” For on-growing purposes and when organic aquaculture juvenile animals are not available, non-organic aquaculture juveniles may be brought into a holding. At least the latter two thirds of the duration of the production cycle shall be managed under organic management (*Article 25e*).

Thus, all the specimens collected for this study, in both intensive and organic facilities, shared standardized intensive conditions (water temperature = ∼10°C; dissolved oxygen = 12 ppm; density: ∼ 13 kg · m^−3^) until they attained the weight of about 10 g. ORG1 fish originated from the same farm where the INT1 lot was sampled and ORG2 from INT2. The ORG3 lot originated in the hatchery of the same farm.

Samples were euthanatized with a lethal dose of 2-phenoxyethanol (0.5 mg/L), frozen and X-rayed (4 min/5 mA/80 kW) in order to perform meristic counts and skeletal anomalies analysis.

Sampling and killing procedures in this study complied with the Institutional Animal Care and Use Committee (IACUC) guidelines.

The vertebral column was divided into four regions, based on distinct morphological features. Vertebrae were split into cephalic (equipped with epipleural ribs), pre-haemal (with epipleural and pleural ribs and open haemal arch, without haemal spine), haemal (with haemal arch closed by a spine) and caudal (with haemal and neural arches closed by a modified, elongated spine; urostyle was included).

The anatomical terminology is according to [44]–[46], except for caudal fin structure terminology, which is according to [47].

The following meristic counts were considered: (1) vertebrae; (2) epural and hypurals; (3) anal and dorsal rays; (4) anal and dorsal pterygophores; (5) principal caudal fin rays, divided into upper (UPCR) and lower (LPCR); (6) supraneural bones.

The correlation between meristic counts and total length (TL) was tested by a Spearman rank correlation. The standard descriptive statistics (median and range) for each meristic count were calculated from the raw data. The significance of the differences in the median values of each meristic count was tested by means of the non-parametric Kruskal-Wallis test, with Mann-Whitney pairwise post-hoc comparisons. ANOSIM (Analysis Of SIMilarities) was applied to the overall matrix of meristic counts to compare intensively vsorganically-reared specimens. ANOSIM is a non-parametric test of significant difference between two or more groups, based on any distance measure [48]. In this study, Euclidean distance was selected for meristic counts. Distances were then converted to ranks. The test is based on comparing distances between groups with distances within groups. Let *r_b_* be the mean rank of all distances between groups, and *r_w_* the mean rank of all distances within groups. The test statistic R is then defined as: R = (r_b_–r_w_)/[N(N-1)/4].

A large positive R (up to 1) signifies dissimilarity between groups. The significance is computed by permutation of group membership (10,000 replicates).

The list of anomalies considered is set out in Table 3. Some anomalies displayed different degrees of alteration (see, for example, C3 and C3* in Table 3) and were indicated as distinctive variables. In this study we chose to distinguish severe anomalies from the biologically severe anomalies as they lead to some commercial (and not only biological) consequences (i.e., unmarketable fish, Table 3): i.e., partial or complete vertebrae fusion is not considered as a commercially severe anomaly if it affects only a few (maximum 3) non-adjacent vertebrae, because this would not influence either growth performance or external shape of the fish. The presence of consecutive fusions involving at least 4 adjacent vertebrae, on the contrary, is likely to stiffen the trunk, so they are considered as commercially and biologically severe anomalies. The presence of deformed vertebrae centra is no longer considered a commercially severe anomaly: the methodology applied actually requires that any axis deviation is considered as an anomaly only if at least one of the vertebrae centra included in the deviation is modified. A deformed centrum leading to axis deviation (kyphosis, lordosis or scoliosis) is scored among commercially severe anomalies, whilst a deformed centrum not involved in axis deviation is considered as a biologically severe anomaly not definitely influencing growth, welfare and health performance.

**Table 3:** List of anomalies considered. Bold font indicates commercially severe anomalies.

**Region**	A	Cephalic vertebrae
	B	Pre-hemal vertebrae
	C	Hemal vertebrae
	D	Caudal vertebrae
	E	Pectoral fin
	F	Anal fin
	G	Caudal fin
	H	First dorsal fin
	I	Second dorsal fin
	L	Pelvic fin
**Types**	**S**	**Scoliosis**
	**SB**	**Saddle back**
	**1**	**Lordosis**
	**2**	**Kyphosis**
	3	Incomplete vertebral fusion
	3*	Complete vertebral fusion
	4	Malformed vertebral body
	5	Malformed neural arch and/or spine
	5*	Extra-ossification in the neural region
	6	Malformed hemal arch and/or spine
	6*	Extra-ossification in the hemal region
	7	Deformed pleural rib
	7*	Extra-ossification of pleural ribs
	8	Malformed pterygophore (deformed, absent, fused, supernumerary)
	9	Malformed hypural (deformed, absent, fused, supernumerary)
	9*	Malformed parahypural (deformed, fused, reduced)
	10	Malformed epural (deformed, absent, fused, supernumerary)
	11	Malformed ray (deformed, absent, fused, supernumerary)
	**12**	**Swim-bladder anomaly**
	13	Presence of calculi in the terminal tract of the urinary ducts
	**14**	**Malformed dentale**
	**15**	**Malformed premaxilla and maxilla**
	**16**	**Dislocation of glossohyal**
	**17sx**	**Deformed or reduced left opercular plate**
	**17dx**	**Deformed or reduced right opercular plate**
	17*sx	Deformed or reduced left branchiostegal ray
	17*dx	Deformed or reduced right branchiostegal ray
	18	Malformed supraneural bones

Vertebrae fusions are considered severe only if affecting at least three consecutive vertebrae.

Paired (pelvic and pectoral) fins were not considered in this study because they were often excessively eroded in the samples examined.

Some assumptions were made in carrying out the analysis: i) non-completely fused bone elements were counted as distinct elements in meristic counts; ii) supernumerary bones with a normal morphology were not considered as an anomaly but as a meristic count variation; conversely, anomalous supernumerary elements were considered anomalies; iii) only the clearly and unquestionably identifiable variations in shape were considered as skeletal anomalies: if any doubts arose, then the shape variation was not considered anomalous; iv) misalignments of vertebrae were considered as lordosis and/or kyphosis only if the vertebral bodies involved were deformed.

The data matrix was processed to calculate skeletal anomaly incidence and to perform a descriptive analysis for each anomaly type and lot.

Anomaly data were then converted into binary values (presence or absence of each anomaly type) and frequencies of specimens affected by each anomaly in each lot were calculated. The resulting matrix (32 skeletal typologies x 8 lots) was then subjected to Correspondence Analysis (CA – [49]), in order to visualize the relationships among lots and the role that each anomaly plays in the ordination model.

ANOSIM was applied to the binary matrix of anomalies to compare intensively and organically-reared specimens, using the Rogers & Tanimoto similarity coefficient [50].

Kruskal-Wallis test, ANOSIM and Correspondence Analysis were performed using PAST (version 2.14 [51]).

## Results

Median and ranges of meristic counts are shown in Table 4. No significant correlation was detected between size (TL) and each meristic count, thus excluding any size effect on the observed meristic counts.

**Table 4:** Median and ranges of meristic counts.

Lot	Tot	Ceph	Pre-hem	Hem	Caud	Ep	Hyp	UPCR	LPCR	An Pter	An Rays	Supr	Do Pter	Do Rays
**1**	63	2	37	17.5	7	3	6	10	9	12	15	18	13	16.5
	62–65	2–3	35–39	15–19	5–8	2–3	5–6	10–11	8–9	11–14	14–17	15–20	12–15	15–18
**2**	63	2	36	18	7	3	6	10	9	13	15	18	13	16
	57–65	1–2	33–38	13–21	6–8	1–3	5–7	9–11	7–10	11–14	14–17	15–20	11–15	13–18
**3**	62	2	35	18	7	3	6	10	9	12.5	16	18	13	16
	60–64	–	34–36	15–19	6–8	2–3	–	–	9–11	11–14	15–17	16–20	12–14	14–18
**4**	63	2	36	18	7	3	6	10	9	12	15	18	13	16
	62–64	2–3	35–37	17–19	7–8	–	5–6	9–11	–	11–13	13–16	16–19	12–14	15–17
**5**	63	2	36	17	7	3	5	10	9	13	16	18	13	16
	59–65	1–3	34–38	16–19	5–9	2–4	5–6	9–11	8–10	11–14	14–17	16–21	11–15	13–18
**6**	62	2	35	18	7	3	6	10	9	12	15	18	13	16
	61–63	1–3	34–36	16–19	7–9	1–3	5–6	–	9–10	11–13	13–17	16–21	12–14	13–16
**7**	62	2	35	18	7	3	6	10	9	12	15	18	13	16
	60–64	–	33–36	16–20	6–9	2–3	–	10–11	8–10	11–14	13–17	15–22	11–14	13–17
**8**	63	2	37	18	7	3	6	10	9	12	15	18	13	16
	60–65	–	35–37	16–20	6–8	2–3	5–6	9–10	8–9	10–14	13–16	16–20	11–14	12–17

Tot: total number of vertebrae; Ceph: cephalic vertebrae; Pre-hem: pre-hemal vertebrae; Hem: hemal vertebrae; Caud: caudal vertebrae; Ep: epurals; Hyp: hypurals; UPCR: upper principal caudal rays; LPCR: lower principal caudal rays; An Pter: anal pterygophores; Do Pter: dorsal pterygophores; Supr: supraneurals.

The total number of vertebrae varied greatly from 57 (French trout reared in INT1) to 65 (several lots reared both under intensive and organic conditions), but median values ranged from 62 to 63. The number of cephalic and caudal vertebrae were the most canalized, with same medians in all lots (2 and 7, respectively); more variation was observed in the median values of haemal (17–18) and pre-haemal (35–37) vertebrae. Epurals, hypurals, UPCR, LPCR and dorsal pterygophores showed no variation in the median values, while anal pterygophores and rays, supraneurals and dorsal rays showed little variation. Significant differences were detected using the Kruskal-Wallis test in the total number of vertebrae (H = 85.17; p<0.0001), pre-haemal vertebrae (H = 152.4; p<0.0001), haemal vertebrae (H = 58.09; p<0.0001), caudal vertebrae (H = 26.55; p<0.0001), epurals (H = 8.47; p<0.001), UPCR (H = 2.05; p<0.05), LPCR (H = 122.2; p<0.0001), anal pterygophores (H = 63.09; p<0.0001) and rays (H = 42.03; p<0.0001), dorsal pterygophores (H = 63.32; p<0.0001) and rays (H = 60.34; p<0.0001). Mann-Whitney post-hoc pairwise comparisons (Bonferroni corrected) are reported in Table 5. ANOSIM detected highly significant differences in meristic counts between intensive and organic lots (R = 0.04; p<0.0001).

**Table 5:** Mann-Whitney post-hoc pairwise comparisons (Bonferroni corrected).

Vertebrae (H = 85.17; p<0.0001)								
	1	2	3	4	5	6	7	8
1		*	*		*	*	*	*
2			*	*		*	*	
3				*	*			*
4					*	*	*	*
5						*	*	
6								*
7								
8								
Pre-hemal Vertebrae (H = 152.4; p<0.0001)								
	1	2	3	4	5	6	7	8
1		*	*	*	*	*	*	
2			*	*	*	*	*	*
3				*	*			*
4						*	*	*
5						*	*	*
6								*
7								*
8								
Hemal Vertebrae (H = 58.09; p<0.0001)								
	1	2	3	4	5	6	7	8
1		*		*				
2					*	*	*	*
3					*			
4					*			
5						*	*	*
6								
7								
8								
Caudal Vertebrae (H = 26.55; p<0.0001)								
	1	2	3	4	5	6	7	8
1								*
2				*	*	*	*	*
3								*
4								*
5								*
6								*
7								*
8								
Epurals (H = 8.47; p<0.001)								
	1	2	3	4	5	6	7	8
1								
2				*	*		*	
3								
4								
5								
6								
7								
8								
UPCR (H = 2.05; p<0.05)								
	1	2	3	4	5	6	7	8
1								
2								
3								
4								
5							*	
6								
7								*
8								
LPCR (H = 122.2; p<0.0001)								
	1	2	3	4	5	6	7	8
1			*	*				
2			*	*			*	
3					*	*	*	*
4					*	*	*	*
5								
6								
7								*
8								
Anal Pterygophores (H = 63.09; p<0.0001)								
	1	2	3	4	5	6	7	8
1		*		*	*			
2				*		*	*	*
3				*				
4					*			
5						*	*	*
6								
7								
8								
Anal Rays (H = 42.03; p<0.0001)								
	1	2	3	4	5	6	7	8
1			*	*	*			*
2				*	*	*	*	*
3				*		*	*	*
4					*			
5						*	*	*
6								
7								
8								
Dorsal Pterygophores (H = 63.32; p<0.0001)								
	1	2	3	4	5	6	7	8
1		*	*	*		*	*	*
2					*		*	
3							*	
4					*			
5						*	*	*
6								
7								
8								
Dorsal Rays (H = 60.34; p<0.0001)								
	1	2	3	4	5	6	7	8
1		*	*	*		*	*	*
2					*			
3					*			
4					*			
5						*	*	*
6								
7								
8								

* p<0.05.

**Figure 1:**
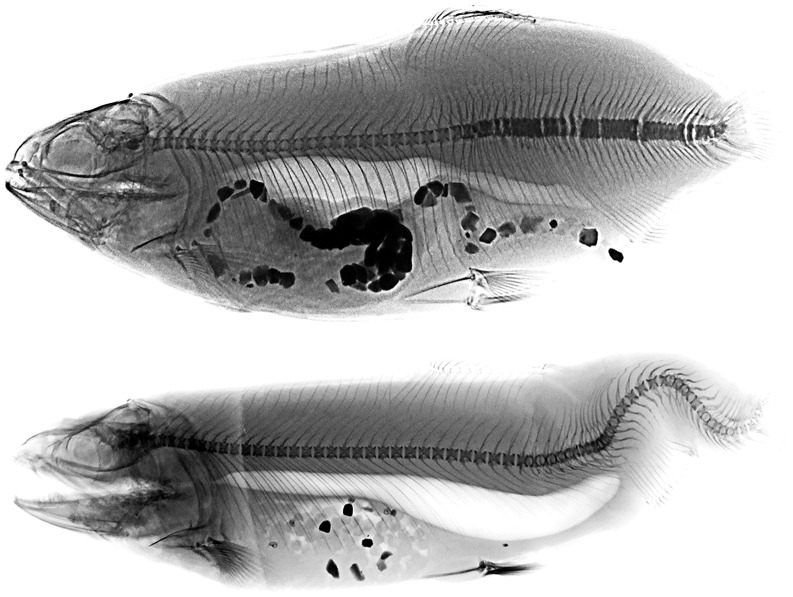
Rainbow trout specimens affected by commercially severe anomalies. Specimen with stumpy body due to scoliosis and compressed hemal and caudal vertebrae, and specimen affected by kypho-lordosis in the hemal and caudal vertebrae. Some hemal vertebrae are compressed and fused.

**Figure 2:**
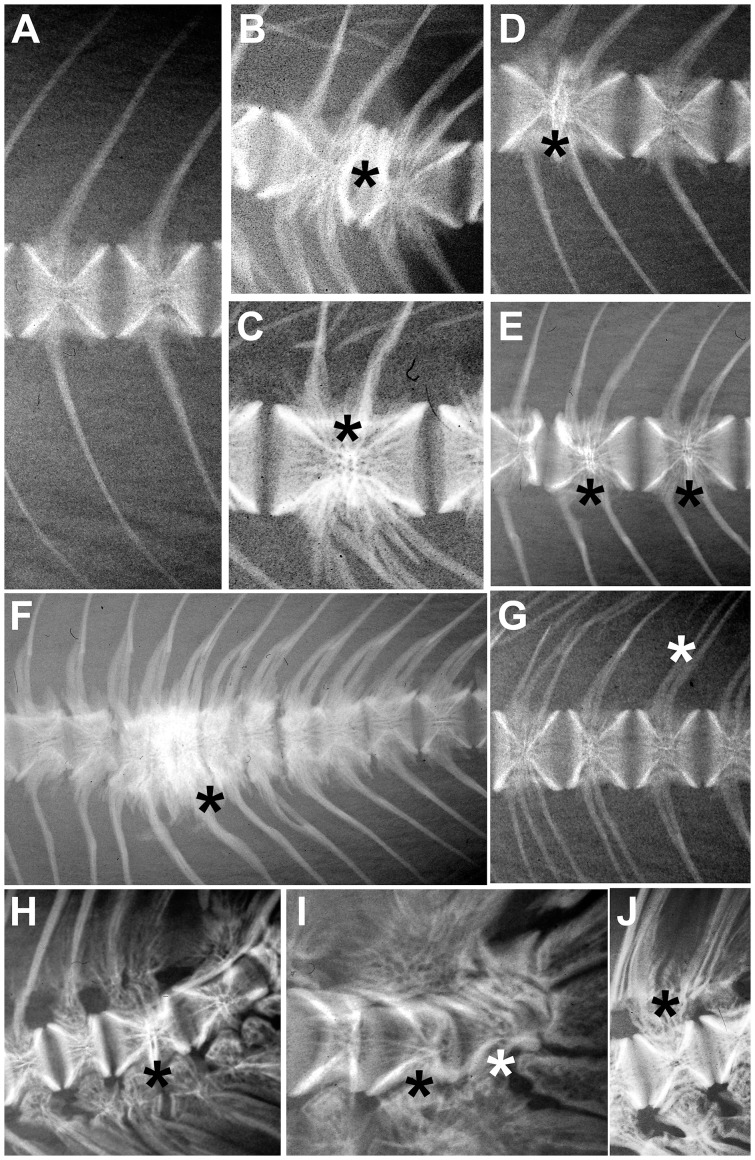
Anomalies observed in rainbow trout specimens. Asterisks indicate the position of the anomalies in the images. A. Normal shaped hemal vertebra; B. one-sided compression of pre-hemal vertebrae (B4), corresponding to type 5 of [58]; C. complete fusion of pre-hemal vertebrae (B3*), corresponding to type 7 of [58]; D. incomplete (C3) fusion of hemal vertebrae; E. complete (C3*) fusion of hemal vertebrae, corresponding to type 7 of [58]; F. compressions and fusions of hemal vertebrae (C3, C3* and C4), corresponding to type 8 of [58]; G. anomalous neural arches (B5); H. incomplete fusion of caudal vertebrae (D3); I. anomalous caudal vertebrae (D4); (J) anomalies of neural spines of caudal vertebrae (D5).

A total of 32 types of anomaly were observed, some of which are shown in Figs. 1 and 2 (A–J). Intensive lots showed inter-lot variations in the anomaly typologies (14–25) than organic lots (20–25; Table 6). Some severe anomalies, affecting the vertebral column (such as scoliosis or saddle back) and the cephalic region (such as the dislocation of the glossohyal, or anomalous opercular plate), as well as swim bladder anomalies and the presence of calculi in the urinary duct were never observed. Some others (C1: kyphosis in hemal vertebrae; B2: lordosis in pre-hemal vertebrae; D2: lordosis in caudal vertebrae) were extremely rare.

**Table 6:** General data on deformed individuals, incidences and typologies of skeletal anomalies in the observed lots.

	1	2	3	4	5	6	7	8
Deformation rate (%)^a^	100	100	100	100	100	100	100	100
Anomaly load^b^	20.3	25.8	26.6	20.7	22.7	24.7	26.7	22.9
No. anomaly types	21	25	20	14	25	22	20	21
Severe anomaly incidence (%)^c^	4.4	0.4	3.8	0.2	2.9	0.1	1.9	0.3
Severe deformation rate (%)^d^	8.7	2.1	12.5	3.1	6.5	3.4	5.0	2.0
Severe anomaly load^e^	2.5	4.5	8.0	1.00	10.3	1.0	10.0	3.0

Organic lots are highlighted with grey background.

a Frequency of individuals with at least one anomaly.

b Total number of anomalies/number of anomalous individuals.

c Number of severe anomalies/total number of anomalies x 100.

d Number of individuals with at least one severe anomaly/total number of lot individuals.

e Number of severe anomalies/number of individuals with severe anomalies.

The percentage of individuals with at least one anomaly was 100% in all lots. The anomalies load was very high, ranging from 20.3 to 26.6 anomalies on each deformed individual in intensive lots, and from 22.7 to 26.7 anomalies on each deformed individual in organic ones. The distribution of the number of anomalies per individual was not normal (Shapiro-Wilk’s test) in both intensively and organically-reared individuals (W_int_ = 0.93, p_int_<0.0001; W_org_ = 0.83, p_org_<0.0001), indicating that in both groups the greatest number of individuals was affected by 15–30 anomalies, with fewer affected by a lower (0–10) or higher (35–70) number of anomalies, and rare individuals characterized by a very high number of malformations (≥ 95) (Fig. 1).

Commercially severe anomalies represented 0.2–4.4% and 0.1–2.9% of the total anomalies inspected in intensive and organic lots, respectively. Intensively reared lots showed higher severe deformation rates, ranging from 2.1 to 12.5% of the individuals versus a load ranging from 1 to10.3% in the semi-intensive individuals. Intensive lot 3 (Italian strain produced from USA eggs and reared in INT2) showed the highest severe deformation rate (12.5%) and severe anomaly load (8.0), while semi-intensive lot 8 (Swiss strain obtained from Germany and reared in ORG3) showed the lowest severe deformation rate (2.0%). The highest and the lowest severe anomaly loads were both observed in/organic lots, i.e. lots 5 (10.3 severe anomalies/individual; Italian trout reared in ORG1) and 6 (1 severe anomaly/individual; Italian strain from USA, reared in ORG2), respectively.

Some types of anomaly were observed only in a few intensively reared lots (Table 7): lordosis in pre-haemal vertebrae (B2), complete fusion between the bodies of the same vertebrae (B3*), or of the caudal vertebrae (D3*). However, kyphosis in haemal vertebrae (C1), lordosis in the caudal vertebrae (D2), and deformed caudal rays (G11) were observed only in organic lots. All these anomalies were very rare and not evenly distributed among the lots.

**Table 7:** Frequency (%) of individuals affected by each anomaly in each lot.

	1	2	3	4	5	6	7	8
**A5**	78.3	1.0	87.5	62.5	64.8	48.3	66.7	71.4
**A3**	6.5	1.0	6.3		0.9	6.9	5.0	2.0
**A4**	10.9		18.8			10.3		2.0
**B2**	2.2							
**B3**		5.2	6.3	3.1	5.6	10.3	8.3	4.1
B3*		1.6						
**B4**	4.3	1.6	18.8		2.8		8.3	2.0
**B5**	100.0	99.5	100.0	100.0	96.3	100.0	98.3	100.0
**B7sx**	80.4	54.9	62.5	46.9	38.9	41.4	40.0	55.1
**C1**					0.9			
**C3**	6.5	1.0			7.4	3.4	1.7	2.0
**C4**		1.0			3.7	3.4	3.3	
**C5**	15.2	38.9	18.8	25.0	19.4	31.0	33.3	34.7
**C6**	39.1	32.1	37.5	46.9	31.5	48.3	48.3	49.0
**D2**					0.9			
**D3**	8.7	2.1	12.5		11.1	3.4	1.7	2.0
D3*		0.5						
**D4**	8.7	4.7	6.3		1.9	3.4	1.7	4.1
**D5**	58.7	40.4	43.8	46.9	54.6	41.4	38.3	38.8
D5*	13.0	22.3	31.3	15.6	25.9	20.7	26.7	20.4
**D6**	39.1	35.8	50.0	21.9	54.6	20.7	31.7	30.6
D6*		1.6	6.3		0.9			
**F8**	10.9	10.9	12.5	9.4	12.0	6.9	13.3	16.3
**F11**		1.0				3.4		
**G9**	2.2	3.6	6.3		2.8	6.9	3.3	8.2
**G10**	15.2	23.8	18.8	9.4	24.1	20.7	11.7	20.4
**G11**								2.0
**H8**	15.2	9.8	6.3	3.1	26.9	17.2	20.0	6.1
**H11**		1.6			1.9			
**18**	56.5	42.0	43.8	34.4	46.3	51.7	56.7	55.1
**14**				3.1		3.4		
**15**	2.2				1.9			
**Abs** a								

Empty cells indicate 0.0%. Organic lots are highlighted with grey background.

a Absence of anomalies.

The most frequent anomaly was B5 (deformation of neural arches and spines in pre-hemal vertebrae – Fig. 2G) in all the observed lots, followed by A5 (deformation of neural arches and spines in cephalic vertebrae) and B7 (deformed pleural ribs). Neural arches and spines of all vertebrae were often anomalous in all lots.

The pre-haemal region of the vertebral column was the most affected by anomalies as it was the only region affected by severe anomalies in all lots, except for lots 4, 6 and 8 (Table 8). Also commonly affected were the cephalic and caudal regions, with no clear pattern of linkage with rearing methodology or strain. Fin anomalies and head malformations were evenly distributed in organic and intensive lots. Head malformations were quite rare (1.9–3.4% of individuals affected).

**Table 8:** Frequency of individuals (%) affected by anomalies/severe anomalies (in bold) in the different body regions. Empty cells indicate 0.0%. Organic lots are highlighted with grey background. VT = vertebrae.

	1	2	3	4	5	6	7	8
**Cephalic VT**	80.4	73.1	100.0	62.5	65.7	62.1	70.0	71.4
**Pre-Hem. VT**	100.0 **2.2**	100.0 **1.0**	100.0 **18.7**	100.0	99.1 **3.7**	100.0	100.0 **1.7**	100.0
**Hemal VT**	50.0	53.9 **0.5**	50.0	56.3	39.8 **5.6**	58.6	65.0 **5.0**	65.3
**Caudal VT**	78.3 **4.3**	70.5 **0.5**	75.0	59.4	77.8 **3.7**	65.5	66.7	59.2 **2.0**
**Anal Fin**	10.9	10.9	12.5	9.4	12.0	10.3	13.3	16.3
**Caudal Fin**	15.2	25.9	25.0	9.4	25.9	24.1	13.3	30.6
**Dorsal Fin**	15.2	11.4	6.3	3.1	26.9	17.2	20.0	6.1
**Head**	**2.2**			**3.1**	**1.9**	**3.4**		

**Figure 3:**
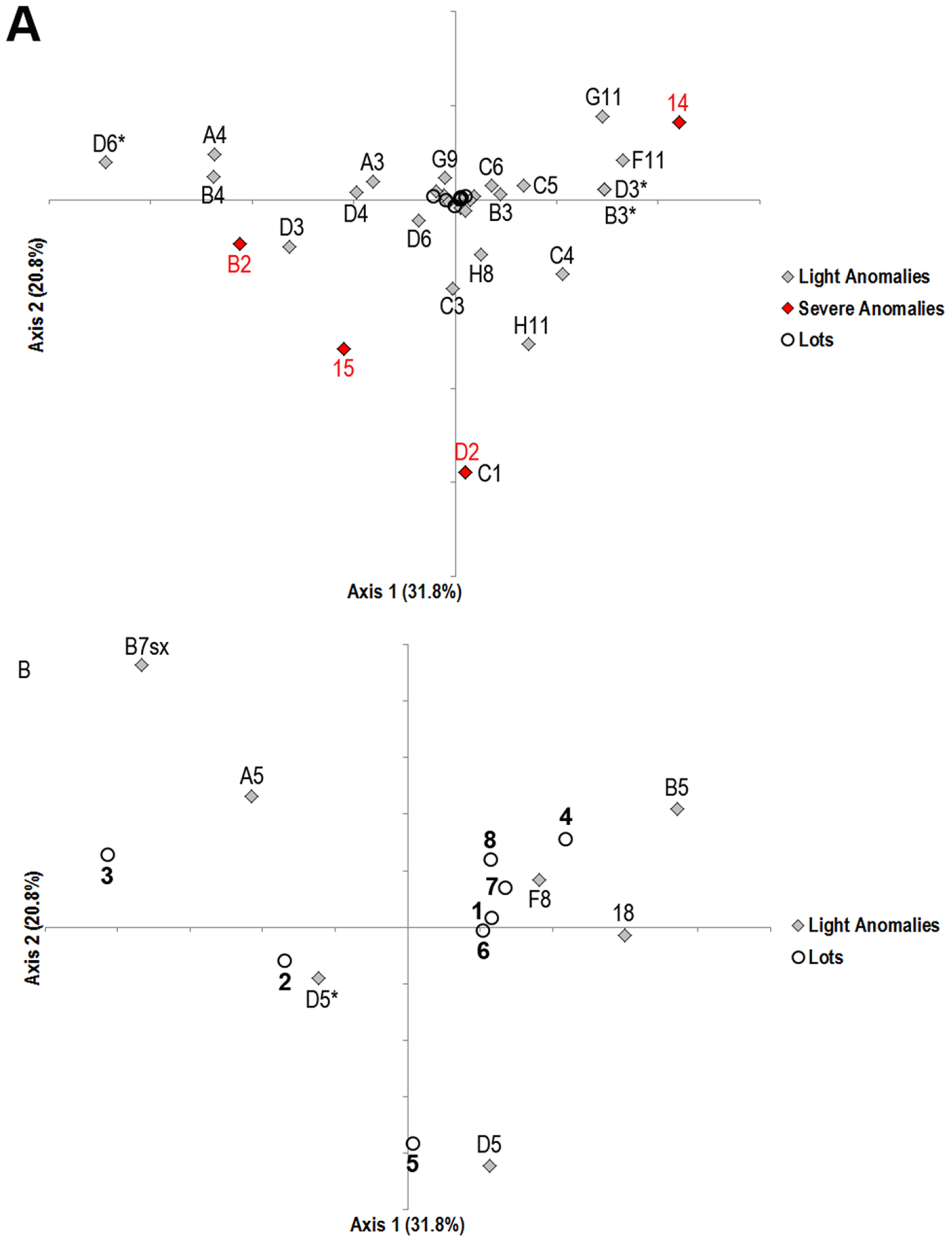
Correspondence Analysis ordination plot. CA (A)ordination of anomalies and lots (axis 1 *vs* 2). Red codes highlight commercially severe anomalies; (B) magnification of (A), in order to visualize lot arrangement more clearly.

The CA ordination plot of lots and descriptors (anomalies) on the first two correspondence axes is shown in Figure 3A and 3B. The first two axes accounted for 31.8% and 20.8% of the overall variance, respectively. As the lot centroids were much closer to the axis origin than most of the descriptor points, the ordination of lots was also plotted on a separate enlarged figure (Fig. 3B), in order to visualize lot arrangements more satisfactorily. Intensive lots were more scattered in the space described by the first two axes compared with the organic ones, which are all located in the negative portion of the first axis. Lots coming from ORG2 (6 and 7) and ORG3 (8) were very close to each other and located in the second quadrant, while the lot sampled in ORG1 (lot 5) was positioned in the third quadrant.

No farm-related patterns (lots coming from the same farm, such as 1 and 2 or 3 and 4, were very far from each other) or related to the genetic origin (e.g., Italian lots were not closer to each other than to the other lots) were clearly detectable.

Anomalies clustered in four main groups (Fig. 3A):

1. anomalies of the vertebral bodies (A4, B4– Fig. 2B – and D4– Fig. 2I) and the presence of extra-ossifications in the haemal arches of the caudal vertebrae (D6*) in the first quadrant, fusions of the cranial (A3) and caudal vertebrae (D3– Fig. 2H), kyphosis in the pre-haemal region (B2) and malformed premaxilla and maxilla (15) in the negative region of CA1;
2. anomalies of the caudal and anal fin rays (G11, F11), complete fusion of pre-haemal vertebrae (B3*–Fig. 2C) and malformed dentale (14) in the second quadrant of the ordination;
3. anomalies of the haemal vertebrae (C1, C3–Fig. 2D–and C4– Fig. 2F), those affecting the second dorsal fin (H8 and H11) and kyphosis in caudal vertebrae (D2) located in the third quadrant;
4. descriptors closer to the axis origin, common to all the observed lots.

ANOSIM detected the mean inter-group distances for lots reared in intensive and organic farms as significant (R = 0.02, p<0.0001). The anomalies thus seemed to be related to the rearing conditions. However, no significant differences between intensive and organic lots were detected (R = 0.0005, p = 0.12) when only severe anomalies were considered.

## Discussion

This study represents one of the first attempts [52] to characterize and compare the skeletal quality of rainbow trout reared under intensive and organic aquaculture. In salmonids, vertebral axis deviations appear dramatically only after smoltification, and are only rarely observed in early juveniles [12]. One exception is that the displacement of vertebral bodies has been reported in under yearling smolts of fast growing intensively-reared salmon [28]. Fin anomalies other than fin erosion are rarely reported in salmonids [12]. Because anomalies are the subject of significant economic [24] and animal welfare concern [53]–[55], it is important to identify their potential causes and find appropriate rearing conditions for ensuring correct skeletal development.

Recent effort to rear rainbow trout under organic aquaculture is an opportunity to analyse if this methodology can produce trout of higher morphological quality than the intensive rearing technology.

In this investigation, several lots of adult rainbow trout from intensive and organic farms were inspected for the presence of skeletal anomalies. Unlike the majority of available studies on rainbow trout [6], [8], [26], [27], [29], [56]–[62], the presence of anomalies affecting the vertebral axis, the unpaired fins, and the *splanchnocranium* were scored. The frequencies of each kind of anomaly in each body region were described, and a detailed computation made of the meristic characters.

The rainbow trout spine normally consists of 59–63 vertebrae [63], even if some previously analysed hatchery lots [64] and wild populations [65], [66] showed wider ranges of variation (the minimum and maximum values reported are 58 and 67, respectively – Table 9). In this study, the number of vertebrae varied from 57 to 65 in intensive lots, and from 59 to 65 in organic ones. Although organically-reared lots show a narrower range of variation, the interquartile distribution of the number of vertebrae was nearly the same for the two groups (61–64 vs. 61–63, respectively). The wider range in the intensive lots was therefore due to the presence of a few outliers. The pre-haemal region was the most variable portion of the vertebral column, with 33–39 and 33–38 elements in intensive and organic lots, respectively. The cephalic and caudal regions were very conservative. Rainbow trout is a subcarangiform generalist swimmer, propelled by the undulatory motion of the body with the caudal peduncle acting as a single unit (BCF) [67]: the whole body is involved in the undulatory propulsion, but wave amplitude is maximum near the tail or in the posterior third of the body. Joined to a relatively deep caudal peduncle, there is a caudal fin characterized by a low aspect ratio [68]. The involvement of the caudal peduncle in the swimming propulsion, i.e. one of the most important and adaptive functions of fish, is probably the reason leading to the high degree of canalization of the number of vertebrae in this region. Conversely, the number of thoracic vertebrae (above all the pre-haemal ones) is probably less strictly controlled, as this region of the body is not directly involved in generating thrust.

**Table 9:** Summary of meristic ranges in previously analysed reared [64] and wild [65], [66] rainbow trout. MX: Mexico; BC: Bogota Columbia; AK: Alaska; AJ: S. Africa Jonkershoek; AP: S. Africa Pirie; SP: Spain; PG: Poland; NS: Normandale Spring; ID: Idaho; NF: Normandale fall.

Origin	Strain	Vertebrae	Dorsal Rays	Caudal Rays	Anal Rays	Source
**Native**		60–66	12–16	18–20	12–16	[65], [66]
**Farmed**	**MX**	58–64	14–17	19	13–16	[64]
	**BC**	60–65	13–17	19–20	13–16	
	**AK**	60–63	13–15	17–19	12–15	
	**AJ**	60–64	13–17	19–20	13–16	
	**AP**	60–64	14–17	18–20	13–16	
	**SP**	60–63	14–18	18–19	13–16	
	**PG**	61–64	14–17	19–20	12–16	
	**NS**	61–65	15–17	18–19	14–16	
	**ID**	63–67	14–16	18–19	13–15	
	**NF**	60–64				

Previous meristic counts in wild and reared rainbow trout revealed range values of dorsal (12–18), caudal (17–20) and anal rays (12–16) that substantially overlapped those recorded in the lots analysed in this study (Table 9– [64]–[66]). Moreover, in the hatchery lots observed by MacGregor & MacCrimmon [64], some of the meristic characters analysed (i.e. vertebrae, anal and dorsal rays) showed significant different mean values as they are useful characters for stock discrimination. In this study, beyond vertebrae and anal and dorsal fin rays, anal pterygophores and supraneurals showed median value differences among lots, corroborating previous investigations.

All individuals displayed at least one anomaly in all lots. Such high rates of anomalous individuals in reared lots of rainbow trout, never previously described in literature, could be explained by applying the methodology applied in this study, which has now been amply standardized and already applied to other farmed, mostly marine, fish [11]–[13], [15], [16], [33]–[36]. The detailed and mass monitoring of all anomalies affecting the *splanchnocranium*, vertebral axis and fins was never applied to salmonids, often scored only for vertebrae centra anomalies, or inspected only for externally detectable anomalies (see Table 10 for a brief review of some studies on salmonids anomalies), of furnishing lower deformation rates. For instance, some authors found that up to 55% of normally shaped rainbow trout (i.e. showing no external anomalies) of market size were found to be affected by vertebral anomalies on French farms [59]. Others reported that a certain number of Atlantic salmon were affected to a different degree by a variable number of compressed vertebrae that were not externally visible [28].

**Table 10:** Summary of some previous studies on salmonid skeletal anomalies. Occurrence refers to the percentage of affected individuals (mean±S.D., range or maximum).

Species	Developmental stage	Types of anomalies considered	Inspection methodology	Occurrence (%)	Source
*O. mykiss*	Juvenile	Vertebral axis	External visual inspection	3–10	[6]
*O. mykiss*	Juvenile	Splanchnocranium, vertebral axis and fins	*In toto* staining	62.8±26.9	[69]
*S. trutta*	Adult	Vertebral axis	External visual inspection	8.9	[8]
*O. mykiss*	Sub-adult	Vertebrae centra	X-rays	9.8±3.1	[70]
*S. salar*	Juvenile and adult	Vertebrae centra	X-rays	0–100*	[71]
*S. salar*	Pre- and post-smolt	Splanchnocranium	External visual inspection	20–65	[9]
*O. mykiss*	Sub-adult	Vertebrae centra	X-rays	50.6	[56]
*S. salar*	Adult	Vertebral axis	External visual inspection	2.3–21.5	[24]
*S. salar*	Embryo	Vertebral axis	Not specified	14	[72]
*S. salar*	Sub-adult	Vertebral axis	X-rays	27–34	[73]
*S. salar*	Adult	Vertebral axis (short-tail phenotype)	X-rays	35	[26]
*S. salar*	Juvenile	Vertebral axis	X-rays	45–60	[74]
*S. salar*	Pre- and post-smolt	Vertebrae centra	X-rays	12	[56]
*S. salar*	Juvenile and smolt	Splanchnocranium and vertebral axis	X-rays	7.0–12.4	[75]
*O. mykiss*	Adult	Splanchnocranium and vertebral axis	External visual inspection	7.1±9.5	[76]
*O. mykiss*	Adult	Vertebrae centra	X-rays	21.1±16.1	[59]
*O. mykiss*	Sub-adult	Vertebrae centra	X-rays	60.0	[27]
*S. salar*	Juvenile	Vertebrae centra	X-rays	33.7**	[60]
*O. mykiss*	Juvenile	Vertebral axis	External visual inspection	10–45	[77]
*S. salar*	Juvenile	Vertebral axis	X-rays	8.9–13.9	[29]
*O. mykiss*	Adult	Rib and vertebrae centra	X-rays	39.3	[78]
*S. salar*	Post-smolt	Vertebrae centra	X-rays	37	[79]
*S. salar*	Juvenile	Vertebrae centra	X-rays	25–92**	[80]
*S. salar*	Post-smolt	Vertebrae centra	X-rays	2.5–16.4	[81]
*S. salar*	Juvenile	Vertebral axis	*In toto* staining	29.6	[82]
*S. salar*	Juvenile	Splanchnocranium and vertebral axis	External visual inspection	<2.5%	[83]

*Percentage of columnal length with changes in *centra.*

**Range/Maximum percentage of anomalous vertebrae, not individuals.

No differences in the occurrence of deformed individuals were detected between intensive and organic conditions. However, ANOSIM found significant mean inter-group distances for lots reared on intensive and semi-intensive organic farms. This is due to differences in the anomaly pattern; intensive lots showed higher inter-lot differences in the anomaly typologies (14–25 types) than the organic ones (20–25 types – Table 6), as highlighted by their scattered distribution in the CA ordination plot with respect to the organic lots (Fig. 3B). The most frequent anomalies were B5 (deformed neural arches and spines in pre-haemal vertebrae) and the presence of extra-ossifications of pleural ribs (B7*). Some anomalies were detected only in organic lots, i.e. anomalies of caudal (G11) and anal (F11) fins rays and axis deviations (scoliosis and kyphosis) of the haemal and caudal region (C1 and D2). All these anomalies were detected in three different semi-intensive lots, so they cannot be ascribed to specific sub-lots of the organic group. In particular, the only individuals affected by C1 and D2 typologies were both detected in the same lot- the Italian lot reared in ORG1 (Lot 5), which is the organic farm characterized by the highest rearing densities and the lowest water renewal. The peculiar pattern of anomalies in the individuals reared on this farm was also emphasized by its isolated position in the CA ordination plot with respect to the ORG2 and ORG3 lots, which were closer to each other.

No clear patterns of skeletal anomalies distinguishing between the different lots on a genetic basis were found. The observed differences in the anomaly typologies and frequencies in the intensive and organic lots were statistically significant (ANOSIM), thus indicating the presence of an effect of rearing methodology on skeletal anomalies, even if a clear pattern characteristic of intensive or organic lots has not been identified.

A non-significant higher average percentage of individuals affected by severe anomalies was detected in intensive lots (6.6% vs. 4.2%). Fused and anomalous cephalic vertebrae (A3 and A4) were absent (A4) or quite rare (A3) in all lots, except for Italian strains, both intensively and organically-reared. Anomalies affecting fin rays were rarer than those involving pterygophores. No clear relationship between the degree of anomaly and the rearing conditions or genetic origin was evidenced.

Dentale, pre-maxilla and maxilla anomalies were found in a few individuals and lots, and were not related to specific rearing conditions. These data suggest that, in rainbow trout, unlike marine reared fish [11], [12], [84]–[88], anomalies affecting skeletal elements other than vertebrae and the vertebral axis are quite rare.

In this study, vertebrae arches and centra were the most commonly affected elements, varying from only a single abnormal vertebra to various compressed and/or fused vertebrae. This reveals a wide range of plastic responses of the salmonid axial skeleton to environmental factors [26], [53], [54], [57], [68], [71], [74], [89]. Previous studies [27], [56], [59] reported caudal vertebrae as being the most likely to be affected by severe anomalies. Also in this study it was common for caudal vertebrae to be anomalous, especially in intensively-reared lots. This is probably due to the sub-carangiform swimming of this species, in which the muscles located in this region ensure propulsion [90]–[93], but also exert strong mechanical forces, which could determine intervertebral joint failures and then vertebrae compression and fusion [26], [57]. Mechanical forces exerted by extra-activity of muscles on the column under intensive rearing conditions may lead to bone and cartilage remodelling, thus generating spinal anomalies. Moreover, stressful handling procedures (e.g., vaccination) in intensive farming conditions could induce inflammation [94], which has been hypothesized to induce bone and/or cartilage remodelling [62] leading to vertebrae compression.

As organic production is based on non-organic aquaculture juveniles, it is necessary to compare adult stages of the same origin in order to analyse whether rearing conditions affect skeletal anomaly pattern and/or occurrence. In this study, only Italian strains had adults both in semi-intensive organic and intensive conditions. This suggests that organic adults showed a larger number of anomaly typologies and a lower ratio of severe anomalies and a lower occurrence of severely deformed individuals compared with the same lot reared in intensive conditions. On close examination (Table 7), it appears that the observed differences are very small, refer to 1–2 individuals, and are a probable consequence of sampling.

The lack of significant differences in the incidence of severe anomalies in intensive and semi-intensive lots, in contrast to what had previously been observed in some reared marine fish (i.e., *Pagrus pagrus* [31], *Sparus aurata* [36]), suggests that factors other than stocking density and water volume influence the skeletogenetic processes in rainbow trout.

The lack of significant differences between rainbow trout adults on-grown under traditional intensive and organic aquaculture could be explained by a variety of hypotheses.

Common conditions shared during embryonic, larval and early juvenile developmental stages could be the most likely cause of such a lack of significant differences in the occurrence of anomalies and in the pattern of severe anomalies. It has been emphasized that spinal anomalies can develop at all life stages of Atlantic salmon [57]. Indeed, several critical stages for the development of bone anomalies have been identified, such as egg incubation, the period between yolk sac alevins and first feeding juveniles, first feeding period to smoltification and later, the seawater period [95]. These results would suggest the need for the establishment of protocols for the organic rearing of larvae and juveniles and for organic broodstocks in order to produce high quality fish. The possibility of introducing non-organic juveniles in organic farms for on-growing will be banned in the next two years (EC 710/2009) thus making it essential for fish farmers to make an effort in this direction.

Another hypothesis that should be considered and tested in the future is the loss of adaptive potential of fully domesticated strains of rainbow trout and the consequent reduced ability to phenotypically react to new environmental cues due to both decreased genetic variability and phenotypic plasticity [96]–[98]. Genetic variability in captive populations is generally subject to intense reduction due both to non-directional (i.e., inbreeding and genetic drift) and directional mechanisms (i.e., artificial selection, reduction of natural selection) [99]–[100]. Loss of genetic variation in hatchery stocks maintained in captivity for a long time has harmful effects on a variety of important traits related to fitness (e.g., survival of eggs and larvae, growth rate, feed conversion efficiency, risk-taking behavior and swimming performance) [101]–[104], thus impairing the ability to adapt to changes in environmental conditions. Adaptive response to changes in environmental conditions may also depend on phenotypic plasticity: the genotype, through interactions with the environment, generates different phenotypes, depending on the external conditions [105]. Historically, environmentally affected phenotypes were scarcely considered because of their apparent lack of a genetic basis. The modern view rejects this notion and, in many circumstances, phenotypic plasticity is considered adaptive. This view can be summarized in the statement that “phenotypic plasticity evolves to maximize fitness in variable environments” [106]. On the basis of this assumption, it could be hypothesized that the constant biotic and abiotic conditions experienced in captive environments make the high maintenance costs of phenotypic plasticity pointless, thus impairing genotype skill to generate different phenotypes under the thrust of changing external cues (a phenomenon denoted as *environmental robustness* [97], [107]-[112], that is to say the insensitivity of the phenotypic outcome to environment). This could be considered as a new kind of homeorhetic trajectory [113], where the fluctuation of physiological variables is stabilized. Very little is yet known, however, about how developmental systems generate robustness when exposed to variation in ecologically relevant conditions [114].
