# Correction: Skeletal Anomaly Monitoring in Rainbow Trout (*Oncorhynchus mykiss*, Walbaum 1792) Reared under Different Conditions

**DOI:** 10.1371/journal.pone.0111294

**Published:** 2014-10-15

**Authors:** The PLOS ONE Staff

There is an error in Table 10. The Occurrence (%) value in line 21 should be 82 ±11. Please find a corrected table below.

**Table 10:** Summary of some previous studies on salmonid skeletal anomalies. Occurrence refers to the percentage of affected individuals (mean±S.D., range or maximum).

Species	Developmental stage	Types of anomalies considered	Inspection methodology	Occurrence (%)	Source
*O. mykiss*	Juvenile	Vertebral axis	External visual inspection	3-10	[6]
*O. mykiss*	Juvenile	Splanchnocranium, vertebral axis and fins	*In toto* staining	62.8±26.9	[69]
*S. trutta*	Adult	Vertebral axis	External visual inspection	8.9	[8]
*O. mykiss*	Sub-adult	Vertebrae centra	X-rays	9.8±3.1	[70]
*S. salar*	Juvenile and adult	Vertebrae centra	X-rays	0-100*	[71]
*S. salar*	Pre- and post-smolt	Splanchnocranium	External visual inspection	20-65	[9]
*O. mykiss*	Sub-adult	Vertebrae centra	X-rays	50.6	[56]
*S. salar*	Adult	Vertebral axis	External visual inspection	2.3-21.5	[24]
*S. salar*	Embryo	Vertebral axis	Not specified	14	[72]
*S. salar*	Sub-adult	Vertebral axis	X-rays	27-34	[73]
*S. salar*	Adult	Vertebral axis (short-tail phenotype)	X-rays	35	[26]
*S. salar*	Juvenile	Vertebral axis	X-rays	45-60	[74]
*S. salar*	Pre- and post-smolt	Vertebrae centra	X-rays	12	[56]
*S. salar*	Juvenile and smolt	Splanchnocranium and vertebral axis	X-rays	7.0-12.4	[75]
*O. mykiss*	Adult	Splanchnocranium and vertebral axis	External visual inspection	7.1±9.5	[76]
*O. mykiss*	Adult	Vertebrae centra	X-rays	21.1±16.1	[59]
*O. mykiss*	Sub-adult	Vertebrae centra	X-rays	60.0	[27]
*S. salar*	Juvenile	Vertebrae centra	X-rays	33.7**	[60]
*O. mykiss*	Juvenile	Vertebral axis	External visual inspection	10-45	[77]
*S. salar*	Juvenile	Vertebral axis	X-rays	8.9-13.9	[29]
*O. mykiss*	Adult	Rib and vertebrae centra	X-rays	82 ±11	[78]
*S. salar*	Post-smolt	Vertebrae centra	X-rays	37	[79]
*S. salar*	Juvenile	Vertebrae centra	X-rays	25-92**	[80]
*S. salar*	Post-smolt	Vertebrae centra	X-rays	2.5-16.4	[81]
*S. salar*	Juvenile	Vertebral axis	*In toto* staining	29.6	[82]
*S. salar*	Juvenile	Splanchnocranium and vertebral axis	External visual inspection	< 2.5%	[83]

*Percentage of columnal length with changes in centra.

**Range/Maximum percentage of anomalous vertebrae, not individuals.
